# A Mouse Model of Nigrostriatal Dopaminergic Axonal Degeneration As a Tool for Testing Neuroprotectors

**DOI:** 10.32607/actanaturae.11433

**Published:** 2021

**Authors:** A. A. Kolacheva, M. V. Ugrumov

**Affiliations:** Koltzov Institute of Developmental Biology of Russian Academy of Sciences, Moscow, 119334 Russia

**Keywords:** Parkinson’s disease, axonal terminal, model, test system, neuroprotector

## Abstract

Degeneration of nigrostriatal dopaminergic neurons in Parkinson’s disease begins from the axonal terminals in the striatum and, then, in retrograde fashion, progresses to the cell bodies in the substantia nigra. Investigation of the dynamics of axonal terminal degeneration may help in the identification of new targets for neuroprotective treatment and be used as a tool for testing potential drugs. We have shown that the degeneration rate of dopaminergic axonal terminals changes over time, and that the striatal dopamine concentration is the most sensitive parameter to the action of 1-methyl-4-phenyl-1,2,3,6-tetrahydropyridine (MPTP). This model was validated using neuroprotectors with well-known mechanisms of action: the dopamine transporter inhibitor nomifensine and SEMAX peptide that stimulates the secretion of endogenous neurotrophic factors or acts as an antioxidant. Nomifensine was shown to almost completely protect dopaminergic fibers from the toxic effect of MPTP and maintain the striatal dopamine concentration at the control level. However, SEMAX, slightly but reliably, increased striatal dopamine when administered before MPTP treatment, which indicates that it is more effective as an inductor of endogenous neurotrophic factor secretion rather than as an antioxidant.

## INTRODUCTION

Investigation of the molecular mechanisms of neurodegeneration and neuroplasticity is the key to understanding the mechanisms underlying normal aging, accompanied by a constant relatively slow loss of neurons (4.5% over 10 years) and the pathogenesis of congenital and chronic nervous system diseases associated with an accelerated loss of neurons [1]. One of the most common neurodegenerative diseases, Parkinson’s disease (PD), is characterized by impaired motor function due to the loss of the dopaminergic (DA-ergic) neurons of the nigrostriatal system. Currently, only symptomatic treatment with dopamine (DA) agonists is used in PD, which does not stop neuronal loss, leading to rapid disability in patients. Attempts to additionally use neuroprotective therapy have not yet been successful. Indeed, drugs with neuroprotective properties, which have shown high efficiency in animal models of Parkinsonism, have not passed clinical trials [2, 3]. This is associated with a critical decrease in the number of DA-ergic neurons by the time of treatment initiation [4]. On the other hand, primary screening of neuroprotective agents in PD models does not consider the dynamics of neuronal degeneration when the test agent is administered either before induced death of DA-ergic neurons or after, stimulating compensatory brain reserves.

Previously, using 1-methyl-4-phenyl-1,2,3,6-tetrahydropyridine (MPTP), we developed a mouse model of the early clinical PD stage, which was used to study the late period of DA-ergic neuronal death and the period of development of compensatory processes. However, the initial period of neurodegeneration development was not investigated. Therefore, the purpose of this study was to develop a model of degeneration of DA-ergic neuronal terminals in the striatum and test potential neuroprotective agents. A comprehensive study of the morphological and functional parameters of axonal terminals in the initial period after MPTP administration identified the parameters most sensitive to the action of neurotoxin.

At the next stage, the developed test system was used to test two known neuroprotectors with different mechanisms of action: nomifensine, an inhibitor of the DA membrane transporter involved in the penetration of specific toxins (MPP+, 6-HDA) into DA-ergic neurons, followed by oxidative stress and subsequent neuronal death; and SEMAX, a fragment of the adrenocorticotropic hormone, a Met-Glu-His-Phe-Pro-Gly- Pro peptide that can act either as an inducer of the synthesis of endogenous neurotrophic factors or an antioxidant, depending on the way of its administration [5, 6].

## EXPERIMENTAL

We used male C57BL/6 mice (2–2.5 months of age). The mice were kept in standard vivarium conditions (free access to food and water and a 12 h day/night cycle). Animal manipulations were performed according to the protocol that was approved by the Bioethics Committee of the Koltsov Institute of Developmental Biology of the Russian Academy of Sciences and was consistent with national and international requirements.

**Fig. 1:**
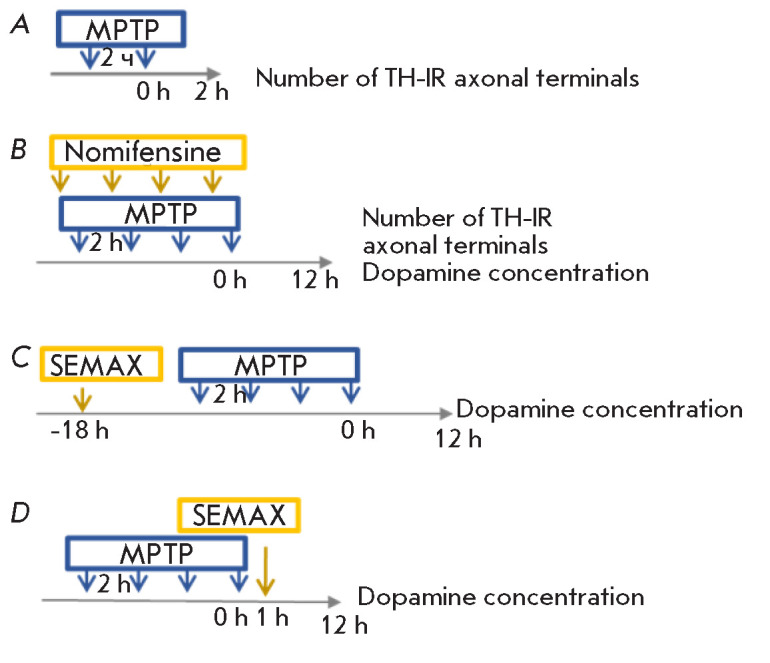
Experiment design. (*A*) The number of TH-immunoreactive axonal terminals in striatal slices and the striatal DA concentration (HPLC-ED) 2 h after two MPTP injections (subcutaneously (s.c.) 12 mg/kg with an interval of 2 h). (*B*) Nomifensine (s.c. 10 mg/kg) was administered 30 min before each of 4 MPTP injections (s.c. 12 mg/kg with an interval of 2 h). Twelve hours after the last MPTP injection, the striatal DA concentration was determined by HPLC-ED and the number of TH-immunoreactive axonal terminals was assessed immunohistochemically in striatal slices. SEMAX (intranasally (i.n.) 50 μg/kg) was administered once either 12 h before the first MPTP injection (*C*) or 1 h after the last MPTP injection (*D*). The striatal DA concentration was determined by HPLC-ED 12 h after the last MPTP injection

The morphological and functional state of DA-ergic axonal terminals in the initial period of their degeneration in the striatum was assessed 2 h after two injections of MPTP (Sigma Aldrich, USA) at a single dose of 12 mg/kg with an interval of 2 h. Control animals were injected with 0.9% NaCl according to a similar scheme (*Fig. 1A*). Tyrosine hydroxylase (TH) (*n *= 3–4) in striatal slices was detected by immunohistochemistry (IHC), followed by the counting of axonal terminals in four areas of the dorsal striatum as described previously [7]. Also, the striatal DA concentration was determined by high-performance liquid chromatography with electrochemical detection (HPLC-ED) (*n *= 5–7) (*Fig. 1A*).

R/S-nomifensine, 1,2,3,4-tetrahydro-2-methyl- 4-phenyl-8-isoquinolinamine (Sigma, USA), was administered subcutaneously at a single dose of 10 mg/kg 30 min before each of four subcutaneous MPTP injections at a dose of 12 mg/kg with an interval of 2 h (*n *= 8–9) (*Fig. 1B*). A Met-Glu-His-Phe-Pro-Gly- Pro peptide (SEMAX) was administered intranasally at a dose of 50 μg/kg, according to two schemes (the agent was provided by the National Research Center “Kurchatov Institute”). The synthesis of endogenous neurotrophic factors was induced by a SEMAX injection 12 h before the first of the four MPTP injections at a dose of 12 mg/kg with an interval of 2 h (*Fig. 1C*); as an antioxidant, SEMAX was administered 1 h after the last of the four MPTP injections at a dose of 12 mg/kg with an interval of 2 h (*Fig. 1D*) (*n *= 5–0). Material (striatum) for all neuroprotectors was collected 12 h after the last MPTP injection, and the DA concentration in the tissue was determined by HPLC-ED. Also, the nomifensine experiment included a quantification of TH-immunoreactive axonal terminals in the striatum. Details of the procedures for immunohistochemical detection of TH, counting of axonal terminals, and measuring of the striatal DA concentration are described elsewhere [7].

The statistical significance of the collected data was assessed using the parametric Student’s *t*-test and nonparametric Mann–hitney U-test. Differences were considered statistically significant at *p * < 0.05; *p * < 0.1 was considered as a trend towards change. Data are presented as a mean Ѓ} standard error of the mean and expressed as a percentage of the controls taken as 100%.

## RESULTS AND DISCUSSION

Degeneration of nigrostriatal DA-ergic neurons in PD begins from the axonal terminals (varicosities) in the striatum and progresses retrogradely to the neuronal bodies [4]. It should be noted that there are few studies that have explored the period of nigrostriatal system degeneration at the striatum level in the early stages after administration of MPTP. Almost all of these studies determined only the optical density per unit area using a semiquantitative immunohistochemical analysis of TH in striatal slices [8, 9], which was interpreted as the degree of axonal degeneration. However, this is not entirely correct because the axonal TH content in PD and disease models changes [10].

**Fig. 2:**
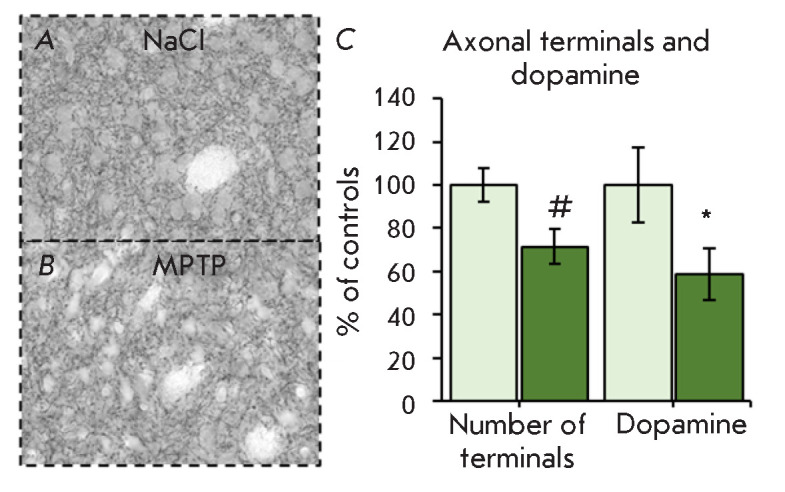
TH-immunoreactive axonal terminals (*A*, *B*), their number, and the DA concentration (*C*) in the striatum 2 h after two MPTP injections (12 mg/kg with an interval of 2 h). * *p* < 0.05, # *p * < 0.1

Previously, we demonstrated that degeneration of DA-ergic axonal terminals in the striatum stops 6 h after four MPTP injections, after which compensatory processes begin to develop (e.g., an increase in the TH activity) [7]. The number of axonal terminals after 3 and 6 h was 67 and 55% of the control value, respectively [7]. In this period, the rate of axonal terminal degeneration within the first hour was 4%. However, this is an indication that the number of terminals at the time of the first MPTP injection should have been about 120%. Therefore, to clarify the rate of nigrostriatal system degeneration within the initial period, we selected a point 2 h after two MPTP injections and found that the number of varicosities was 72% of the control value (*Fig. 2 A–C*). Comparing these data, it appears reasonable to conclude that the rate of loss of axonal terminals is not linear: it is about 7%/h within the first 4 h after the first MPTP injection and 1%/h during the next 5 h.

The nonlinear pattern of axonal terminal degeneration may be associated with the metabolism of MPP+ (a toxin formed from MPTP in glial cells) that is absorbed by DA-ergic neurons using DAT and induces oxidative stress. Once inside a neuron, MPP+ competes with DA for loading into vesicles via the vesicular monoamine transporter 2 (VMAT2), which is a mechanism that protects neurons from death.

The high rate of axonal terminal degeneration up to 2 h after the second MPTP injection is probably associated with the initiation of oxidative stress by MPP+ and the inability to neutralize it by accumulation in DA-filled vesicles. The neurotransmitter is gradually released and degraded, and the striatal DA concentration amounts to 59% 2 h after two MPTP injections (*Fig. 2B*). At the next stage, the rate of axonal terminal degeneration abruptly decreases due to an established balance between the uptake of MPP+ into vesicles and the ongoing release of DA with its degradation.

Therefore, a model of DA-ergic axonal terminal degeneration for testing potential neuroprotectors should primarily focus on the striatal DA concentration as the indicator most sensitive to the action of MPTP. However, given the nonlinear pattern of DA-ergic axonal terminal degeneration, the actual period of neuroprotective action is limited to 6 h after induction of nigrostriatal system neurodegeneration.

At the next stage, we evaluated the possibility of using the dynamics of axonal terminal degeneration as a test system for drugs with neuroprotective properties. For this purpose, we used two neuroprotective agents possessing the “direct” (selective) and “indirect” effects. The direct effect is inhibition of neurotoxin uptake through DAT. Indeed, along with MPTP, there are other neurotoxins that can selectively enter DA-ergic neurons and cause oxidative stress: e.g., salsolinol that forms from DA and can be captured by DAT [11].

**Fig. 3:**
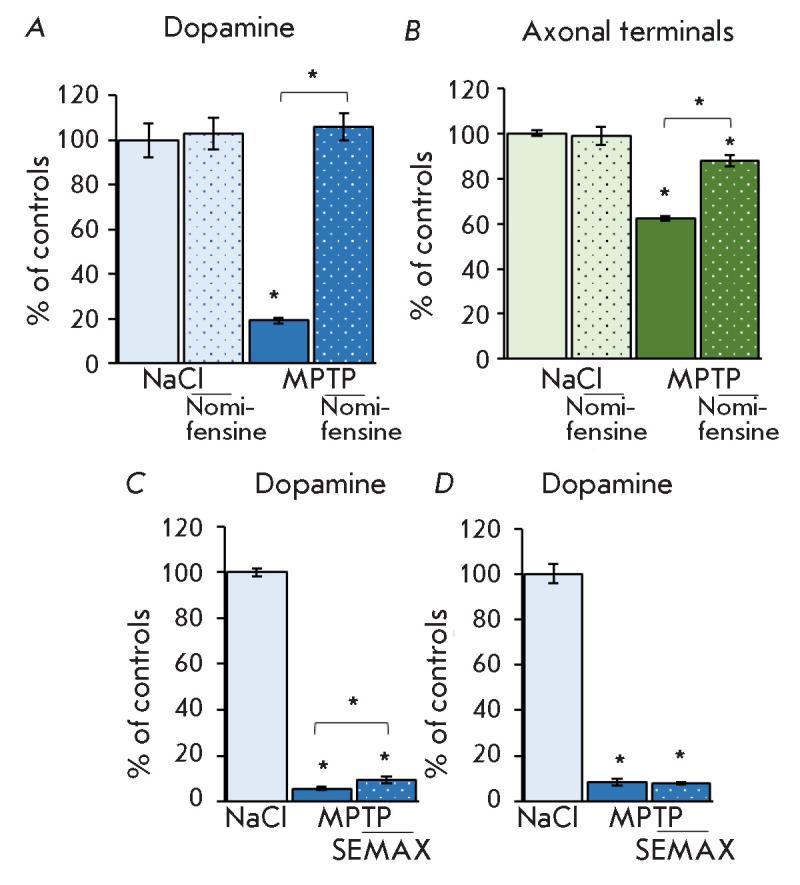
The DA concentration (*A*) and the number of TH-immunoreactive axonal terminals (*B*) in the striatum 12 h after four MPTP injections (12 mg/kg) and a nomifensine injection (10 mg/kg) 30 min before each MPTP injection. The striatal DA concentration 12 h after four MPTP injections (12 mg/kg) and a SEMAX injection (50 μg/kg) 12 h before the first MPTP injection (*B*) or 1 h after the last MPTP injection (D). * *p * < 0.05

Administration of nomifensine was shown to maintain the striatal DA concentration at the control level upon MPTP treatment and significantly protect axonal terminals (*Fig. 3 A,B*). Furthermore, given that uptake of MPP+ occurs through DAT, its inhibition by nomifensine is also the “reference” for the action of potential neuroprotectors.

SEMAX can stimulate the production of endogenous neurotrophic factors and act as an antioxidant [5, 6]. To separate these two effects, we used two experiment designs. In the first case, SEMAX was administered 12 h before MPTP to increase the expression of endogenous neurotrophic factors, or 1 h after the last MPTP injection. An increase in DA was observed only in the group receiving SEMAX 12 h before MPTP (*Fig. 3 C,D*). Also, this group showed a significant decrease in DA turnover (DOPAC/DA) compared to the group receiving MPTP alone (data not shown). Given that SEMAX does not affect the striatal DA level [12], the obtained data indicate a neuroprotective effect of SEMAX on DA-ergic neurons; however, to enhance this effect, the experiment design should be altered; e.g., through use of multiple injections of the agent.

## CONCLUSIONS

Thus, we may conclude that the most sensitive indicator of the effectiveness of the neuroprotector action is the striatal DA concentration, which reflects biochemical changes. In the case of a positive effect on the neurotransmitter level, it is necessary to focus on organic changes in the striatum by counting the DA-ergic axonal terminals. Also, the dynamics of DA-ergic neuronal terminal degeneration may be used as a test system for assessing the effectiveness of neuroprotectors.

## Abbreviations

DA: dopamine
DA-ergic neuron: dopaminergic neuron
DAT: dopamine transporter
HPLC-ED: high performance liquid chromatography with electrochemical detection
IHC: immunohistochemistry
MPP+: 1-methyl-4-phenylpyridinium
MPTP: 1-methyl-4-phenyl-1,2,3,6-tetrahydropyridine
PD: Parkinson’s disease
TH: tyrosine hydroxylase
VMAT2: vesicular monoamine transporter 2.
